# Type 2 diabetes and its correlates among adults in Bangladesh: a population based study

**DOI:** 10.1186/s12889-015-2413-y

**Published:** 2015-10-19

**Authors:** Muhammad Abdul Baker Chowdhury, Md Jamal Uddin, Hafiz M. R. Khan, Md Rabiul Haque

**Affiliations:** Department of Biostatistics, Robert Stempel College of Public Health & Social Work, Florida International University, 11200 SW 8th Street, Miami, FL 33199 USA; Department of Statistics, Shahjalal University of Science & Technology, Sylhet, 3114 Bangladesh; Department of Public Health, Texas Tech University Health Sciences Center, Lubbock, TX 79430 USA; Department of Population Sciences, University of Dhaka, Dhaka, 1000 Bangladesh

**Keywords:** Type 2 diabetes, Correlates, Population based study, BDHS, Bangladesh

## Abstract

**Background:**

Type 2 diabetes is one of the most prevalent non-communicable diseases in Bangladesh. However, the correlates of type 2 diabetes among adults in Bangladesh remain unknown. We aimed to investigate the correlates of type 2 diabetes among the adults in Bangladesh.

**Methods:**

We conducted a cross-sectional study using data from the nationally representative 2011 Bangladesh Demographic and Health Survey. A random sample of 7,543 (3,823 women and 3,720 men) adults of age 35 years and older from both urban and rural areas, who participated in the survey was included. Diabetes was defined as having a fasting plasma blood glucose level of ≥ 7 mm/L or taking diabetes medication during the survey. Hypothesized factors, e.g., age, sex, education, place of residence, social status, body mass index, and hypertension were considered in the analyses. Multivariable logistic regression models were used to identify the important correlates of type 2 diabetes.

**Results:**

Among the respondents, the overall prevalence of diabetes was 11 %, and the prevalence was slightly higher in women (11.2 %) than men (10.6 %). Respondents with the age group of 55–59 years had higher odds of having diabetes (odds ratios (OR) = 2.37, 95 % confidence interval (CI): 1.76–3.21) than the age group of 35–39 years. Moreover, respondents who had higher educational attainment (OR = 1.67, 95 % CI: 1.18–2.36) and higher social status (OR = 2.01, 95 % CI: 1.50–2.70) had higher odds of having diabetes than the respondents with no education and lower social status, respectively. We also found socioeconomic status, place of residence (rural or urban), regions of residence (different divisions), overweight and obesity, and hypertension as significant correlates of type 2 diabetes in Bangladesh.

**Conclusions:**

Our study shows that older age, higher socioeconomic status, higher educational attainment, hypertension, and obesity were found to be significant correlates of type 2 diabetes. Need-based policy program strategies including early diagnosis, awareness via mass media, and health education programs for changing lifestyles should be initiated for older age, wealthy, and/or higher educated individuals in Bangladesh. Moreover, area-specific longitudinal research is necessary to find out the underlying causes of regional variations.

## Background

Diabetes is one of the most prevalent and serious non-communicable diseases (NCDs) all over the world. It is the leading cause of death, disability, and economic loss, and, thus, it is identified as a major threat to global development [1–4]. Moreover, it can lead to a multitude of complications, such as heart disease, stroke, renal failure, and blindness [5–7]. Therefore, it possesses a major health care burden all over the world [8]. Similar to the developed countries, several studies [3, 9–13] suggested that people of Asia, especially South East-Asia (SEA) are at a higher risk of type 2 diabetes irrespective of region, diet, and socioeconomic status [14]. According to the International Diabetes Federation (IDF) [3], the SEA region consisting of Bangladesh, India, Sri Lanka, and Nepal is the home of more than 72 million adults with diabetes, which is expected to exceed 135 million by 2035 [3, 15, 16]. Among the adults (age 20–79 years) with diabetes in the top five SEA countries, Bangladesh is in the second position [3]. The number of people with diabetes in Bangladesh was 5.10 million in 2013, which is expected to increase to 8.20 million (13 % of the total adults) by 2035 [3, 17]. A systematic review and meta-analysis between 1995 and 2010 showed that the prevalence of diabetes among the adults in Bangladesh has increased significantly, 4 % in 1995–2000, 5 % in 2001–2005, and 9 % in 2006–2010 [12]. Although diabetes is a silent killer, nearly half of the population with diabetes is undiagnosed. Furthermore, among those diagnosed with diabetes, only 1 in 3 patients is treated and roughly 1 in 13 achieve treatment targets [18].

Several studies on diabetes have been conducted in Bangladesh [10, 19–22]. However, these studies were small-scale, confined to urban - rural communities or some other specific groups (e.g., slum residents), which did not demonstrate the wide range of correlates of diabetes for the whole country. Therefore, the objective of this study was to identify the correlates of type 2 diabetes using data from the 2011 Bangladesh Demographic and Health Survey (BDHS).

## Methods

### The survey and data source

The Demographic Health Survey (DHS) was designed to collect data to monitor and evaluate population, health, and nutrition status of developing countries [23]. In Bangladesh, this survey has been carried out continuously in a three year interval since 1993 under the authority of the National Institute for Population Research and Training (NIPORT) of the Ministry of Health and Family Welfare. The data files were released in the MEASURE DHS website [23], which are free and available for research. The survey followed the MEASURE DHS model questionnaire and was adopted for use in Bangladesh after a series of meetings with local and international experts [24]. The ICF International located in Calverton, Maryland provided the technical assistance, and the financial support was provided by the United States Agency for International Development (USAID) [24]. Each sequential series of this cross-sectional survey is a nationally representative sample of non-institutionalized population. In this study, we used the data that was collected in 2011, which is the latest available data of the BDHS.

### Sampling design and sample size

**Fig. 1:**
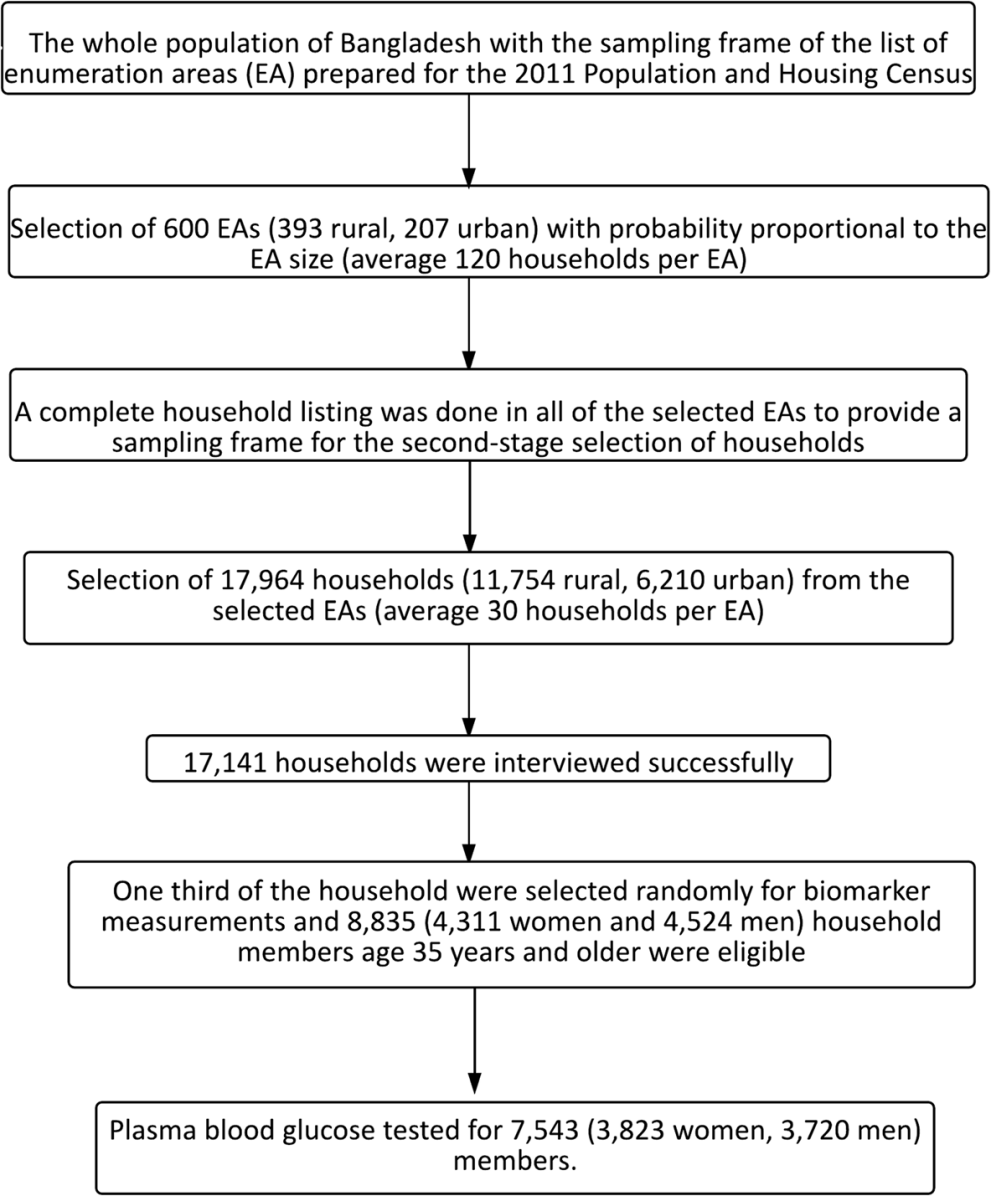
Study population and sample selection

The 2011 BDHS used two-stage stratified cluster sampling from non-institutionalized individual households [24]. The sampling frame used for the survey was the complete list of enumeration areas (EA) covering the whole country of the most recent population census prepared by the Bangladesh Bureau of Statistics (BBS) [25]. An EA is a geographic area covering on average 113 households [24]. In the first stage, 600 EAs (207 urban, 393 rural) were selected with probability proportional to the EA size. In the second stage of sampling, a systematic sample of 30 households on average was selected from each sampling unit to provide statistically reliable estimates of key demographic and health variables for the country as a whole, for urban and rural areas separately, and for each of the seven divisions of Bangladesh. With this design, the survey selected 17,964 (11,754 rural, 6,210 urban) residential households. Among the selected households, 17,141 were interviewed successfully with a response rate of 98 % [24]. For measuring biomarker information, a random subsample (one-third of the households) was selected. In this subsample, all women and men of ages 35 years and older were eligible to participate in the biomarker component, which included blood pressure measurements, testing for anemia, blood glucose testing, and height and weight measurements. A total of 8,835 (4,524 men and 4,311 women) household members of ages 35 years and older from 83,731 household members was included in the subsample [24]. Among them, 92 % of women and 86 % of men participated in the blood pressure measurement, and 89 % of women and 83 % of men participated in the blood glucose measurement [24]. After excluding the missing data and non-responses, the final sample size became 7,543. The sample design and sample selection process is presented in Fig. 1. The detailed survey procedure, study method, and questionnaires are available in the final report of 2011 BDHS [24].

### Study variables

Information on socioeconomic and demographic characteristics of the respondents were collected including age, sex, education, marital status, working status, division of residence, place of residence (rural or urban), wealth status, and anthropomorphic characteristics like blood glucose level, blood pressure, history of diabetes and hypertension, and medication. Some indicators were determined through the physical examination. Height and weight were measured to calculate body mass index (BMI). The BMI was categorized into two categories: normal (BMI ≤ 24.99), overweight and obese (BMI ≥25). Blood pressure and blood glucose were measured by trained health technicians [24].

### Measurements and diagnostic criteria for disease

HemoCue 201+ blood glucose analyzer was used to measure the blood glucose [24]. The survey used the World Health Organization (WHO) [26] cut-off points for measuring fasting plasma blood glucose. An individual was defined as ‘diabetic’, if fasting plasma glucose level was ≥7.0 mmol/L or taking medication to reduce the blood sugar, ‘pre-diabetic’ if fasting plasma glucose level was 6.0–6.9 mmol/L, and ‘diabetes free’ if fasting plasma glucose was below 6.0 mmol/L [26]. For our analysis, we merged two groups (‘pre-diabetic’ and ‘diabetes free’) together to make the variable dichotomous (diabetic patient and non-diabetic patient). The 2011 BDHS used the American Heart Association (AHA) [27] guidelines for cut-off points for blood pressure measurements. An individual was considered as hypertensive if systolic blood pressure (SBP) ≥ 140 mmHG and/or diastolic blood pressure (DBP) ≥ 90 mmHG and/or taking anti-hypertensive medication to reduce blood pressure. Pre-hypertension was defined by SBP ≥120 mmHg but < 140 mmHg and/ or DBP ≥ 80 mmHg but < 90 mmHg and/ or no anti-hypertensive medication at the time of survey. Individuals with SBP and DBP ≤ 120/80 were considered as normal [27].

### Statistical analysis

We conducted bivariate analysis to compare the diabetic respondents across the categories for each of our study variables. A chi-square test was performed to determine the proportional differences by diabetes status. The correlates of diabetes among the adults were assessed by using multivariable logistic regression models. Odds ratios (OR) with 95 % confidence intervals (CI) for correlates of diabetes were estimated. The first model included all selected socio-demographic and economic characteristics, the second model included anthropometric characteristics, the third and the fourth model included interaction terms and square term of age, respectively, and the final model included significant predictors (*p* < 0.05) based on all four models. For selecting the best model, the value of -2LogLikelihood ratio test, AIC, and the area under the receiver operating characteristic (ROC) curve was examined. The lower values of -2Log Likelihood ratio test and lower AIC value represent the better model. The area under the ROC curve measures the accuracy of the model. The summary of modelling exercise is presented in the Appendix (Table 5). An area of 1.0 represents the perfect test and an area of 0.5 represents the worthless test. Appropriate sample weights were used for the whole data set provided by the 2011 BDHS. We used SPSS 20.0 (SPSS, Inc) to analyze the data [28].

## Results

**Fig. 2:**
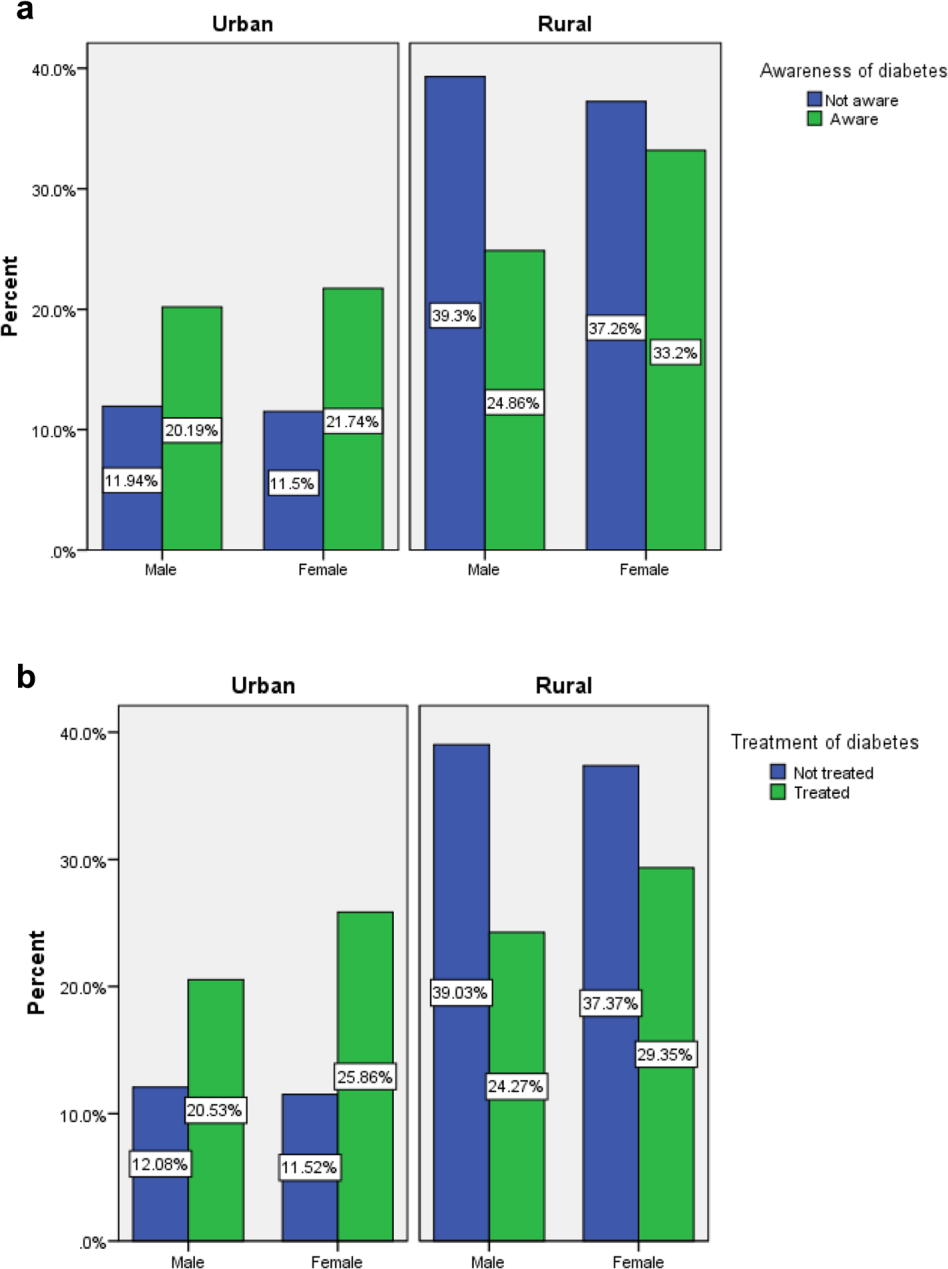
Awareness and treatment status of diabetes by sex and place of residence, Bangladesh Demographic and Health Survey (BDHS), 2011. **a** Awareness of diabetes **b** Treatment of diabetes

A summary of the socioeconomic, demographic, and anthropometric characteristics of the diabetic and non-diabetic respondents was shown in Table 1. Among the respondents (*n* = 7,543), 49.3 % were male and 50.7 % were female. Respondents with older age and higher education had higher percentages of having diabetes compared to the respondents with a younger age and no education. It was also found that most of the diabetic respondents came from the richest households (39.1 %), followed by richer households (21.4 %), and the other wealth index categories had a similar proportion of diabetes (around 13 %). The correlates of diabetes differ significantly with the change in BMI and hypertension. Diabetes was more likely to occur among the study participants with higher body weights (*p* <0.01). Twenty six percent of the diabetic respondents were overweight and obese, and about 65 % of the hypertensive respondents had diabetes. Women and men of ages 35 years and older were asked questions related to any previous diagnosis of diabetes and whether they were taking medication to treat their diabetes. Figure 2 shows the awareness and treatment status of diabetes by sex and by place of residence. The awareness and treatment status among males were more or less similar in both rural and urban areas. However, females in the rural areas were less aware, and a lower proportion of them were taking medicine to control diabetes. Table 2 shows the method of medication of the diabetic patients. Seventy five percent of those receiving treatment take medication orally, 17.39 % take injections, and 7.60 % take medication both orally and by injection. Table 3 shows the distribution of education, body mass index, and hypertension status by place of residence. The urban respondents were found to have higher BMI (26.9 % vs 5.4 %) and be more hypertensive (62.7 % vs 50.2 %) compared to the rural respondents. Table 4 shows the odds ratios and confidence intervals from multivariable logistic regression analysis for different correlates of type 2 diabetes. Participants with increased age and having higher education, higher socioeconomic status, hypertension, and higher BMI were more likely to have type 2 diabetes. Individuals aged 55–59 years had more than two times the chance (OR = 2.37, 95 % CI: 1.76–3.21) of having diabetes than the individuals aged 35–39 years old. In addition, respondents with higher education were 1.67 (95 % CI: 1.18–2.36) times more likely to have diabetes compared to the respondents with no education. The analysis also indicated that individuals who were employed were less prone (OR = 0.74, 95 % CI: 0.58–0.95) to have diabetes compared to those who were not engaged with any work. The wealthier respondents were twice as likely (OR = 2.01, 95 % CI: 1.50–2.70) to be diabetic compared to the lowest income group (reference group) of the population. The odds of having diabetes between overweight and obese persons was found to be 1.83 (95 % CI: 1.51–2.23) compared to the normal weight respondents, and the result was highly statistically significant. Individuals having hypertension were 1.41 (95 % CI: 1.19–1.66) times more likely to have diabetes than the individuals who does not have hypertension. Neither sex nor marital status was strongly associated with having diabetes. A wide variation in the correlates of diabetes was found among the respondents of seven administrative divisions of Bangladesh. Study participants from Barisal, Chittagong, and Dhaka divisions had higher odds of having diabetes compared to the study participants from the Sylhet division.

**Table 1:** Socioeconomic, demographic, and anthropometric characteristic of the study participants by diabetes status, Bangladesh Demographic and Health Survey (BDHS), 2011

	Total		Non diabetic		Diabetic		*p-*value
Variables	*n*	%	*n*	%	*n*	%	
Age group							<0.001
35–39	1415	18.8	1295	19.3	120	14.5	
40–44	1320	17.5	1196	17.8	124	15.0	
45–49	1167	15.5	1037	15.4	130	15.7	
50–54	1013	13.4	907	13.5	106	12.8	
55–59	668	8.9	554	8.2	114	13.8	
60–69	1057	14.0	928	13.8	129	15.6	
70+	904	12.0	799	11.9	105	12.7	
Sex							0.402
Male	3720	49.3	3324	49.5	396	47.9	
Female	3823	50.7	3393	50.5	430	52.1	
Marital status							0.159
Currently married	6355	84.3	5673	84.5	682	82.6	
Others (divorced/separated/widowed)	1188	15.7	1044	15.5	144	17.4	
Education level							<0.001
No education	3581	47.5	3282	48.9	299	36.2	
Primary education	2459	32.6	2164	32.2	295	35.7	
Secondary education	1066	14.1	909	13.5	157	19.0	
Higher education	437	5.8	362	5.4	75	9.1	
Working status							0.001
Not currently working	3933	52.2	3456	51.5	477	57.7	
Currently working	3607	47.8	3257	48.5	350	42.3	
Wealth index							<0.001
Poorest	1472	19.5	1365	20.3	107	12.9	
Poorer	1438	19.1	1332	19.8	106	12.8	
Middle	1493	19.8	1379	20.5	114	13.8	
Richer	1561	20.7	1384	20.6	177	21.4	
Richest	1579	20.9	1256	18.7	323	39.1	
Place of residence							<0.001
Urban	1761	23.3	1478	22.0	283	34.2	
Rural	5782	76.7	5238	78.0	544	65.8	
Division of residence							<0.001
Sylhet (Eastern)	429	5.7	378	5.6	51	6.2	
Chittagong (Southeastern)	1256	16.7	1077	16.0	179	21.6	
Dhaka (Central)	2457	32.6	2180	32.5	277	33.5	
Khulna (Western)	992	13.2	920	13.7	72	8.7	
Rajshahi(Mid-western)	1082	14.3	966	14.4	116	14.0	
Rangpur(Northwestern	898	11.9	820	12.2	78	9.4	
Barisal (Southern)	428	5.7	374	5.6	54	6.5	
Body mass index							<0.001
Normal	6321	86.5	5732	88.1	589	73.6	
Overweight/ obese	985	13.5	774	11.9	211	26.4	
Hypertension							<0.001
No	3533	46.8	3243	48.3	290	35.1	
Yes	4010	53.2	3474	51.7	536	64.9

**Table 2:** Method of medication of diabetes patients by sex, Bangladesh Demographic and Health Survey (BDHS), 2011

	Male		Female		Total	
Medication type	*n*	%	*n*	%	*n*	%
Injected	22	17.9	26	17.0	48	17.4
Orally	95	77.2	112	73.2	207	75.0
Injected and orally	6	4.9	15	9.8	21	7.6

**Table 3:** Distribution of education, BMI, and hypertension by place of residence, Bangladesh Demographic and Health Survey (BDHS), 2011

	Total		Urban		Rural		*p*-value
Variables	*n*	%	*n*	%	*n*	%	
Education level							<0.001
No education	3582	47.5	584	33.2	2998	51.8	
Primary education	2458	32.6	562	31.9	1896	32.8	
Secondary education	1067	14.1	381	21.6	686	11.9	
Higher education	436	5.8	233	13.2	203	3.5	
Body mass index							<0.001
Normal	6322	86.5	1,252	73.1	5070	90.6	
Overweight/ obese	985	13.5	460	26.9	525	5.4	
Hypertension							<0.001
No	3533	46.8	656	37.3	2877	49.8	
Yes	4010	52.2	1105	62.7	2905	50.2

**Table 4:** Estimates of odds ratios and confidence intervals of different correlates of diabetes, Bangladesh Demographic and Health Survey (BDHS), 2011

Variables	OR	95 % CI
Age group		
35–39	1.00	
40–44	1.12	(0.85–1.47)
45–49	1.36	(1.03–1.79)^**^
50–54	1.36	(1.02–1.82)^**^
55–59	2.37	(1.76–3.21)^***^
60–69	1.74	(1.29–2.35)^***^
70+	1.56	(1.10–2.20)^**^
Sex		
Male	1.00	
Female	0.83	(0.65–1.08)
Marital status		
Currently married	1.00	
Others (divorced/separated/widowed)	1.08	(0.85–1.37)
Education level		
No education	1.00	
Primary education	1.32	(1.09–1.60)^**^
Secondary education	1.49	(1.15–1.93)^**^
Higher education	1.67	(1.18–2.36)^**^
Working status		
Not currently working	1.00	
Currently working	0.74	(0.58–0.95)^**^
Wealth index		
Poorest	1.00	
Poorer	0.88	(0.66–1.17)
Middle	0.86	(0.65–1.15)
Richer	1.19	(0.90–1.57)
Richest	2.01	(1.50–2.70)^***^
Place of residence		
Urban	1.00	
Rural	0.98	(0.82–1.22)
Division of residence		
Sylhet (Eastern)	1.00	
Barisal (Southern)	1.90	(1.51–2.37)^**^
Chittagong (Southeastern)	1.63	(1.31–1.97)^***^
Dhaka (Central)	1.42	(1.04–1.94)^**^
Khulna (Western)	0.54	(0.36–0.80)
Rajshahi (Mid-western)	1.04	(0.70–1.47)
Rangpur(Northwestern)	0.80	(0.54–1.19)
Body Mass Index		
Normal	1.00	
Overweight/ obese	1.83	(1.51–2.23)^***^
Hypertension		
No	1.00	
Yes	1.41	(1.19–1.66)^***^

Notes: *** *p* value < 0.001, **p value < 0.05

## Discussion

Our study shows that older age, higher education, affluent socioeconomic status, hypertension, and obesity are significant correlates of type 2 diabetes among the adult population in Bangladesh. We found that respondents between ages 55–59 years have a higher odds of having diabetes compared to those ages 35–39 years. Due to the decline in fertility level and a steady increase in life expectancy in Bangladesh, the population age structure is changing. The number of old age population will increase rapidly, which will strengthen the correlates of diabetes among older age population in the near future. Moreover, diabetes occurs much more among the respondents with higher educational attainment and higher social status. These findings are found to be consistent with previous studies conducted in Bangladesh [10, 22, 29–32] and some neighboring countries [11, 33]. Furthermore, the degree and extent of correlates of type 2 diabetes varies by level of socioeconomic status and income. For example, studies demonstrate that the higher socioeconomic status plays a major role in the reduction of diabetes in the developed countries [34–36], whereas in Bangladesh, we found an inverse relation between higher educational attainment and having diabetes. This result is in line with the other studies of developing countries [34], especially in Asia [37, 38]. We also found that individuals having a higher educational attainment were more likely to have type 2 diabetes compared to less educated individuals. Similar findings were also observed in studies conducted in Bangladesh [29, 30], China [11, 39], and India [33]. Individuals living in urban areas are more likely to have higher BMI and higher odds of having diabetes compared to the individuals residing in rural areas. For example, the overweight or obese individuals are 1.83 times more likely to have diabetes compared to normal weight individuals. Similar findings were found in a study among secretariat employees of Bangladesh [21]. Rahim et al. [10] and Sayed et al. [30] also found that weight gain is a significant predictor of type 2 diabetes among Bangladeshi populations. In contrast, studies conducted by Hussain et al. found a minor correlation between type 2 diabetes and higher BMI for both men and women in 2005 [20] and 2007 [20, 40]. Hence, our study, as well as several previous studies, confirmed that being overweight and obese is the independent and strongest correlate of type 2 diabetes, irrespective of gender, race, and region [32, 41–47]. Several authors showed that for one unit of increase in BMI there is a possibility of increase of diabetes by 12 % [43]. Educated and wealthy individuals may be used to living in urban areas, consuming more fast food, and fatty foods, as well as participating less in physical exercise, which may be the important causes of being overweight and obese, prolonging the correlate of diabetes [48]. The rural people are more likely to be engaged in daily household and other labor intensive activities, and burn a lot of calories that may keep them physically active and lead to lower BMI. Moreover, urban people have insufficient facilities to have physical exercise and to maintain a healthy life which leads them to have higher BMI and therefore, more likely to have diabetes in low and middle income countries [40].

Type 2 diabetes was found to be associated with hypertension. This finding is consistent with cross sectional studies in Bangladesh [21, 22, 40], India [49], China [39], Taiwan [50], and Nigeria [51]. Two population based studies also found that untreated high blood pressure has been linked to diabetes [52]. However, some studies also found the coexistence of hypertension and diabetes [53, 54] across different ethnic, racial, and social groups.

The correlates of diabetes also varied by the regions of residence in Bangladesh: adults from Barisal (Southern), Chittagong (Southeastern), and Dhaka (Central) divisions were more likely to have diabetes compared to the respondents from Sylhet (Eastern) division. There may be limited facilities of physical activity available in these divisions, and individuals are unwilling to take those advantages due to huge road traffic and public safety issues. Since the reasons for these variations are still unknown, longitudinal cohort studies may be conducted to examine the underlying causes behind this variation among the divisions of Bangladesh.

### Strength and limitations

Our study provides evidence of the correlates of type 2 diabetes using the nationally representative sample with comprehensive information on diabetes as well as associated demographic and anthropomorphic characteristics. To our knowledge, this is the first survey in Bangladesh, which collected information on diabetes for adult population by using WHO recommended methods. Therefore, the findings are more representative compared to the previous small scale studies in the country. Despite these strengths, there are several limitations to our study. The findings are generalizable only for the adult populations in developing countries. Apart from the correlates of type 2 diabetes mentioned here, there are a significant number of factors, such as insulin resistance, race or ethnic background, family history of diabetes [30, 55], dieting habits, life style, physical activity, cholesterol level, and cigarette smoking etc., which may be associated with diabetes [39, 56]. We could not include these factors because they were not available in the 2011 BDHS data. Future work should be considered addressing these factors in Bangladesh.

## Conclusions

Consistent with the literature, we found that there is a wide range of factors, which are significantly correlated with type 2 diabetes among the adults in Bangladesh. The findings demonstrate that individuals with older age, higher socioeconomic status, higher education, high BMI, and hypertension have a significant influence on the odds of having diabetes. Moreover, a significant proportion of the adult men and women are not aware of the consequence of this disease, and a small proportion of them are taking medication to control their blood sugar. Since diabetes is a modifiable disease, several recommendations and policy implications can be made based on our study findings. Country-wide diabetes screening programs could be implemented for early diagnosis and control of diabetes with special attention to the older age individuals. These screening programs may help to reduce long-term health complications and the financial cost for the care of the disease. We found that educated and wealthy individuals are more likely to have diabetes in our study. Therefore, motivational programs (i.e., adopting a healthy lifestyle, changing dietary habits, managing blood pressure level, and reducing body weights) should be implemented through specific public health interventions for the wealthy and/or individuals with higher educational attainment. Finally, multi-sectoral preventive strategies including health education programs, especially incorporating information on correlates of type 2 diabetes in the text curriculum at secondary and higher secondary level to build awareness of the disease, mass media campaign to promote physical activity, healthy dieting, and lifestyle changing is essential in Bangladesh.

## Appendix

**Table 5:** ᅟ

Models	Variables	−2 Log likelihood	AIC	H-L Goodness-of-Fit Test (Pr > ChiSq)	AUC (ROC)
Model 1	Sociodemographic and economic characteristics: age, sex, marital status, education, working status, wealth quantile, place of residence, region of residence	Intercept Only = 5213.292	Intercept Only = 5215.292	0.1127	0.67
		Intercept and	Intercept and		
		Covariates =4926.142	Covariates =4976.142		
Model 2	Anthropometric characteristics: hypertension, body mass index	Intercept Only = 5051.305	Intercept Only = 5053.305	0.1692	0.61
		Intercept and	Intercept and		
		Covariates =4909.086	Covariates =4915.086		
Model 3	Model with square of age : age square, sex, marital status, education, working status, wealth quantile hypertension, body mass index, place of residence, region of residence	Intercept Only = 5051.305	Intercept Only = 5053.305	0.3319	0.67
		Intercept and	Intercept and		
		Covariates =4745.731	Covariates =4789.731		
Model 4	Model with interaction terms age, sex, marital status, education, working status, wealth quantile hypertension, body mass index, place of residence, region of residence residence*education, residence*BMI, residence*hypertension	Intercept Only = 5053.305	Intercept Only = 5051.305	0.3691	0.68
		Intercept and	Intercept and		
		Covariates =4776.559	Covariates =4712.559		
Model 5	Final model (Socio demographic, economic, and anthropometric characteristics): age, sex, marital status, education, working status, wealth quantile hypertension, body mass index, place of residence, region of residence	Intercept Only = 5051.305	Intercept Only = 5053.305	0.3789	0.68
		Intercept and	Intercept and		
		Covariates =4713.467	Covariates =4767.467		

## Abbreviations

AHA: American Heart Association
AUC: Area under the curve
BBS: Bangladesh Bureau of Statistics
BDHS: Bangladesh Demographic and Health Survey
BMI: Body mass index
CI: Confidence interval
DHS: Demographic Health Survey
DBP: Diastolic blood pressure
IDF: International Diabetes Federation
NCD: Non-communicable diseases
NIPORT: National Institute for Population Research and Training
OR: Odds ratio
ROC: Receiver operating characteristic
SBP: Systolic blood pressure
SEA: South East-Asia
USAID: United States Agency for International Development
WHO: World Health Organization
